# Functionalizing Van der Waals materials by shaping them

**DOI:** 10.1038/s41377-022-00900-x

**Published:** 2022-07-06

**Authors:** Deep Jariwala

**Affiliations:** School of Engineering and Applied Sciences, University of Pennsylvania, Philadelphia, PA 19104 USA

**Keywords:** Optical properties and devices, Photonic devices

## Abstract

A number of van der Waals materials can be gradually tuned from electron to hole conductance with an increasing or decreasing thickness, which offers a novel route to modulate nanoscale charge-carrier distribution and thus functionality in devices.

The past decade has seen an explosion of research interest in atomically thin materials, typically those bonded via Van-der-Waals forces, due to their intriguing physical properties, such as topology^1^, superconductance^2,3^, valley spin^4^, moiré excitons^5^, ferromagnetic and antiferromagnetic states^6^, bulk-^7^ and flexo-^8^ photovoltaic response. This class of materials also have unique advantages in logic computing^9^, memory storing^10^, polarization^11^, and multicolor photodetection^12^, which promises the solution of (opto-) electronics devices at the ultimate thickness limit.

However, to put those intriguing functionalities into industry-use/reality, one critical issue has to be addressed — a general approach to dope two-dimensional (2D) semiconductors to enable homogeneous junction applications^13^.

Laser^14^, chemical^15^ and surface-transfer^16^ doping techniques where laser illumination is used to produce donor- or acceptor-like defects, chemical dopants (metal atoms, like Cu and Co) are intercalated into the vdWs gap, oxidant and reductant species are used to capture or inject electrons by a surface reaction, respectively have been demonstrated in the past. But, as is well documented in literature, these techniques are mostly customed designed for some specific layered materials, that cannot extend to the whole van der Waals materials family^14–16^.

Recently, writing in this issue of *Light: Science & Applications*, Hui Xia and colleagues at the Shanghai Institute of Technical Physics, Nantong University and Shanghai-Tech University in China report that a variety of van der Waals semiconductor materials (MoS_2_, WSe_2_, MoTe_2_, black-phosphorus) don’t need artificial, external doping and are capable of doping themselves from electron (n) to hole (p) type doping conductance with increasing or decreasing thickness^17^. As schematically shown in Fig. 1, monolayer MoS_2_ is typically n-doped, while multilayer counterpart turns to p-doped. Considering that the self-doping materials span from elemental-layered-semiconductors such as black phosphorus to transition-metal sulfides, selenides, and tellurides, the observed and reported phenomenon might apply to many other layered materials significant expanding their applicability and scope in device design.

**Fig. 1:**
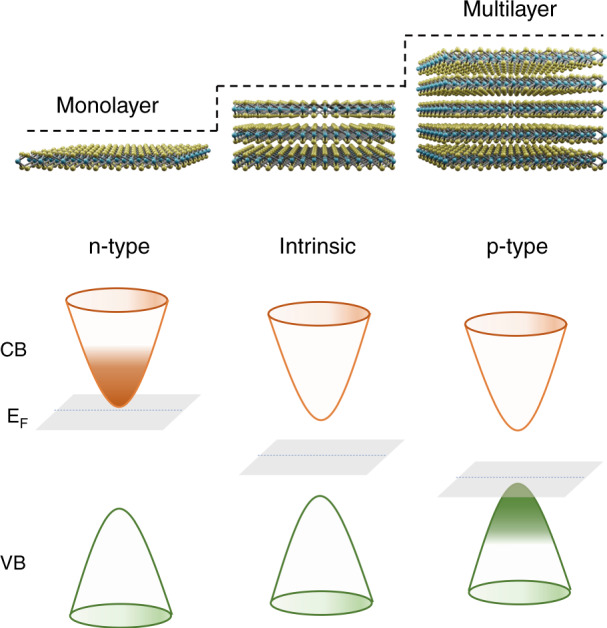
Schematically showing the thickness dependent doping characteristic of layered materials

In such a framework, every monolayer-step change in thickness can ideally serve as a finely tunable knob to spatially modulate the charge-carrier polarity and doping concentration. Further, the geometric boundary serves as a sharp barrier for carrier concentration change. This doping framework therefore offers an opportunity to fabricate atomically abrupt conduction channels and junction. Note that such devices can be very difficult to fabricate by conventional approaches due to uncontrolled dopant interdiffusion process^18^.

The self-doping behavior could benefit the semiconductor manufacturing process of 2D devices, since one only needs to focus on the geometrical morphology, while the van der Waals materials depending on their thickness will address distributing the electrons and holes as required. In the present work the authors have developed and demonstrated a variety of devices based on this concept such as diodes, solar cell and avalanche photodetector.

In the future, more efforts are needed to attain a fundamental understanding of the doping mechanism. What are the factors that drive few- and multilayer- layers to an opposite polarity of doping? Similarly, what is impact of contact fabrication scheme and source material in determining doping type and concentration as a function of thickness? These are outstanding questions they need further investigations. Likewise, for practical realization, attempts for large scale device fabrication with controlled doping as a function of thickness are equally important that will ultimately determine how far can this new doping concept can go.
